# Toxicity Assessment of PEG-PCCL Nanoparticles and Preliminary Investigation on Its Anti-tumor Effect of Paclitaxel-Loading

**DOI:** 10.1186/s11671-018-2615-1

**Published:** 2018-08-24

**Authors:** Wei Li, Wanyi Li, Yu Kuang, Ting Yang, Jie Zhu, Zilin Xu, Xiang Yuan, Mingyuan Li, Zhongwei Zhang, Yuan Yang

**Affiliations:** 1 Department of Microbiology, West China School of Basic Medical Sciences and Forensic Medicine, Sichuan University, Chengdu, 610041 China; 2 Department of Burns surgery, Sichuan Academy of Medical Sciences and Sichuan Province People’s Hospital, Chengdu, 610072 China; 3 Department of Microbiology, Guizhou Medical University, Guiyang, 55000 China; 4 ICU, West China Hospital, Sichuan University, Chengdu, 610041 China

**Keywords:** Toxicity assessment, Poly (ethylene glycol) carboxyl-poly (ε-caprolactone) (PEG-PCCL), Drug delivery/release, Anti-tumor effect

## Abstract

The efficiency of single treatment of conventional chemotherapy drugs is unpleasantly reduced by the physiological barriers of tumors. In this regard, nanoparticles have become attractive for achieving such medical purpose of targeted cancer therapy by delivering anti-tumor agents to the needed area. A novel drug deliverer, poly (ethylene glycol) carboxyl-poly (ε-caprolactone) (PEG-PCCL), has been reported to be highly hydrophilic and stable, while little is known about its organic toxicity. This study focused on systemic toxicity assessments of PEG-PCCL. The pharmacokinetics of PTX-loaded PEG-PCCL (PEG-PCCL/PTX) and its anti-tumor effect were preliminarily investigated. In the present work, PEG-PCCL was characterized by laser particle size analyzer and transmission electron microscopy. The cytotoxicity was investigated by MTT test, LDH leakage assay, immunofluorescence, and transmission electron microscopy. Hemolysis, phlebitis, and organ toxicity tests were performed to demonstrate the biocompatibility and acute biotoxicity. H22 tumor-bearing mice were used to evaluate the pharmacokinetics of the micells of PEG-PCCL/PTX and its anti-tumor effect. The results showed that the size of PEG-PCCL nanospheres was 97 ± 2.6 nm. PEG-PCCL treatment showed little cytotoxicity and good biocompatibility, and did not exhibit organ toxicity. PTX-loading efficiency was 49.98%. The pharmacokinetic study on H22 tumor-bearing mice revealed that PEG-PCCL/PTX has higher stability and slower release than PTX alone. Together, these results suggest that PEG-PCCL nanosphere has little toxicity to organisms and is a potential candidate of biocompatible drug vehicle for hydrophobic drugs.

## Introduction

The rising tendency of cancer incidence is continuing along with the raising of aging population in recent decades [1]. The effectiveness of conventional chemotherapy of cancers has been limited as only a small portion of total dose reaches the tumor site, the remainder of which is distributed throughout healthy tissues, resulting in negative effects especially neutropenia and cardiomyopathy [2]. Nanoparticles represent a potential platform for the delivery of chemotherapeutic drugs due to their unique physical and chemical characteristics [3]. As a result, reduced side effect and enhanced therapeutic efficacy can be achieved. Copolymers based Poly (ethylene glycol) (PEG) and methoxy poly(ethylene glycol) (MePEG)/poly(ɛ-caprolactone) (PCL) are supposed to be promising organic nanoparticles used in drug delivery systems (DDSs), and have already been approved by FDA. These nanoparticles possess easy controlled characteristics like biocompatibility, biodegradability, and thermosensitivity [4]. Some diblock and triblock polymers have been investigated in biomedical applications, like PCL nanosphere [5], PEG-PCL-PEG [6–8], and PCL-PEG-PCL [9] hydrogel. The PCL blocks compose a hydrophobic core capsuling hydrophobic drugs, whereas the PEG blocks form a hydrophilic shell, which makes core-shell micellar nanostructures. These diblock and triblock polymers draw considerable attentions due to the characteristics such as stable structure, prolonged duration in blood circulation, and passive targeting by means of enhanced permeation and retention effect [10]. However, controversial challenges of organic polymers still exist, including toxicity, low drug payloads, undesired drug leakage, and clearance by reticuloendothelial system [11–13].

In comparison with the abovementioned polymers, PEG-PCCL, one that is additionally carboxyl covalently modified on the caprolactone and has been prepared and characterized in our previous studies [14, 15], shows higher hydrophilicity and better stability via the effect of the hydrogen bond. Apart from the physicochemical characteristic results, few data were reported involving in vivo and in vitro toxicity study of polymeric carriers. Nevertheless, predictive models and validated standard methods require a set of design rules involving the toxicity assay of nanoparticles.

Given that, we focused here on in vivo and in vitro acute toxicity assessment of PEG-PCCL qualitatively and quantitatively despite its favorable attributes of high tolerance and biodegradability in vivo. An extensively used nanoparticle in biomedical research, Polyetherimide (PEI), was elected as positive control. Paclitaxel (PTX) is a first-line anti-cancer drug [16], especially an optimized chemotherapeutic in ovarian cancer and non-small cell lung cancer, and has been listed in the *World Health Organization’s List of Essential Medicines*. With the development of nanotechnology, PTX loading in nanoparticles is considered to be potential solution for site-specific drug delivery under the circumstance of multidisciplinary cooperation treatment [17, 18]. In this study, PTX-loaded PEG-PCCL was used to examine the pharmacokinetics and its anti-tumor effect in vivo in hepatic H22 tumor-bearing mice model.

## Methods

### Materials, Cells, and Animals

ε-caprolactone (ε-CL, Alfa Aesar, USA), poly(ethylene glycol) (PEG, Mn = 1000, Fluka, USA), hexamethylene diisocyanate (HMDI, Aldrich, USA), Palladium sur charbon (pd/c, Sigma, USA), Dulbecco’s modified Eagle’s medium (DMEM, Hyclone, USA), 3-(4,5-dimethylthiazol-2-yl)-2,5-diphenyl tetrazolium bromide (MTT, Sigma, USA), bovine serum albumin (BSA, BR, BoAo Co. Ltd., China) were used without further purification. All the materials were analytic reagent grade.

Male Balb/C mice (7–8 weeks old, 20–25 g weight) and New Zealand rabbit (2.5–3.0 kg weight) were purchased from the Chengdu DaShuo biotech Companies (Sichuan, China) with Certificate of Quality No. SCXK2013–24. The animals were maintained in a standard specific pathogen-free environment with sufficient food and tap water. The experiments were conducted according to the Guide for the Care and Use of Laboratory Animals (Ministry of Science and Technology of China, 2006). All the animal experimental procedures were approved by the Ethical Committee for Laboratory Animals of West China Medical Center of Sichuan University.

Mouse H22 hepatocarcinoma cells (H22), human embryonic kidney cells (HEK293T), and hepatoma carcinoma cells (Hep G2) were obtained from Department of Immunology, West China School of Basic Medical Sciences and Forensic Medicine, Sichuan University. HEK293T and Hep G2 were cultured in Dulbecco’s modified Eagle media (DMEM) (Hyclone, UT, USA), supplemented with 10% fetal calf serum (FCS) (Hyclone, UT, USA), and antibiotics (penicillin 100 U/mL and streptomycin 100 U/mL) at 37 °C in 5% CO2.

### Preparation of PEG-PCCL and PTX-Loaded PEG-PCCL Micells

**Fig. 1:**
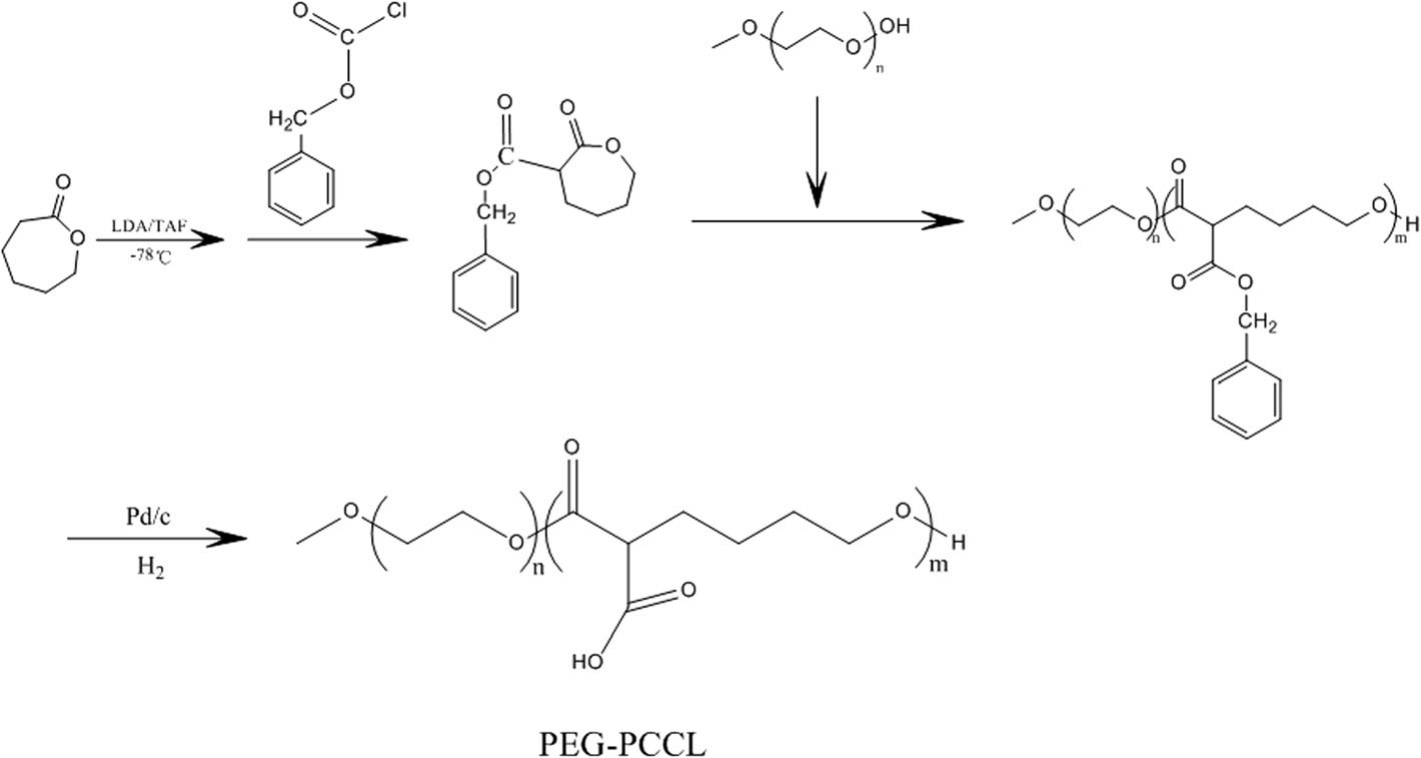
The flow diagram of synthesizing the PEG-PCCL dipolymer

PEG-PCCL and PTX-NPs were supplied by our cooperator, Professor Liu from School of Microelectronics and Solid-state Electronics, University of Electronic Science and Technology of China. The PEG-PCCL diblock copolymers were synthesized by the ring opening polymerization of ɛ-CL in the presence of PEG homopolymer with the catalysts of Palladium sur charbon as the flow diagram below (Fig. 1). The obtained PEG-PCCL copolymers were purified and kept in air-tight bags until use.

To load PTX in PEG-PCCL nanoparticles, solid dispersion, a simple and valuable technique without poisonous organic solvent [19] was performed after PEG-PCCL was prepared. Then, the solution was evaporated at 60 °C under reduced pressure. After the evaporation of alcohol, homogenous copolymers were obtained. PTX was encapsulated by polymeric carriers as amorphous form. The coevaporation was dissolved in water at 65 °C to produce PTX-NPs solution, which was filtered with a 0.22 nm filter to gain a clarified and sterile solution. PTX-NPs powder was got from the lyophilized solution from a freeze dry system. The entrapment efficiency (EE) of PTX was determined with minicolumn centrifugation method [20]. The concentrations of PTX incorporated in PEG-PCCL (C_I_) or the total drug in PEG-PCCL dispersions (C_T_) were analyzed by HPLC. EE (%) = (C_I_ / C_T_) × 100%.

### Characterization

The particle size and zeta potential of PEG-PCCL were measured with laser particle size analyzer (Malvern Nano-ZS 90). Transmission electron microscopy (TEM, H-6009IV, Hitachi, Japan) was used to assess the morphology of PEG-PCCL. Specifically, the solution of nanoparticles was placed on a copper grid covered with nitrocellulose, and then samples were stained with phosphotungstic acid and dried at room temperature.

### Cytotoxicity Assays

The cytotoxicity was assessed by MTT and LDH leakage assay in Hep-G2 and HEK293T. MTS metabolization was quantified in intact cells according to the procedure of MTT [21]. Cell suspensions were prepared by trypsin/EDTA (HyClone, UT, USA). A total of 0.4 × 10^4^ cells were seeded in 96-well plate (Corning, MA, USA). The plates were incubated for 12 h to allow the cells to attach to the plastic surface of the culture plates. Then, the medium was removed and 200 μl fresh culture medium or medium containing different concentrations of PEG-PCCL (from 0 to 1 mg/mL) were added to the wells. No FCS was added to the medium during the 24-h exposure period. The survival rate was determined by cytotoxicity parameters, and was presented by the equation: Survival rate (%) = (OD_T_/OD_C_) × 100. Here, OD_T_ and OD_C_ refer to the absorbance value (measured with the spectrophotometer reader at 570 nm) of PEG-PCCL or PEI nanoparticles treated cells and of untreated cells, respectively.

LDH leakage in culture medium was examined using the LDH assay kit (Biotech, China). All the spectrometric measurements of nanoparticle-treated groups were corrected by a cell-free control. For the morphological study, HepG2 cells was seeded and exposed in the same way as in the cytotoxicity assays. Cells were fixed in 4% paraformaldehyde and were observed under the transmission electron microscope (TEM).

### Apoptosis Assays

Apoptosis was determined by Annexin V-FITC and PI double staining [22]. Briefly, HepG2 cells were seeded in 12-well plates at a density of 4 × 10^4^cells/well and treated with PEG-PCCL (0.5 mg/mL) and PEI (0.5 mg/mL) for 48 h. Then, the cells were washed with cold phosphate-buffered saline (PBS) three times followed by Annexin V-FITC incubation for 15 min and PI staining for another 15 min at 4 °C in the dark. The stained cells were observed under fluorescence microscope (Olympus Co. Ltd., Tokyo, Japan) within 30 min.

### Hemocompatibility Assay

The hemocompatibility of PEG/PCCL was evaluated according to in vitro red blood cell (RBC) hemolysis test reported previously [23]. The mice blood samples were collected, and the erythrocytes were dissolved in PBS (2% RBC solution). Physiologic saline, PEI, or PEG-PCCL of 0.5 mg/ml were mixed with the 2% RBC solution. A positive hemolysis control was prepared by adding an equal volume of erythrocyte suspension and distilled water. After the mixture was maintained for 1 and 3 h at 37 °C and then centrifuged at 2000 r/min for 5 min, the supernatants were detected with a microplate reader (Bio-Rad, CA, USA) at 570 nm. The percentage of hemolysis was calculated by the equation: hemolysis % = (OD_T_–OD_NC_)/(OD_PC_–OD_NC_) × 100. Here, OD_T_, OD_NC_, and OD_PC_ refer to the absorbance values of sample, negative control, and positive control, respectively. Moreover, the in vivo hemolysis was assayed by counting RBC number from the blood sample collected through caudal vein of the mice treated with normal saline, PEI (20 mg/kg), or PEG-PCCL (20 mg/kg) for 3 h.

### Rabbit Phlebitis

Veins in both ears of rabbits were employed to compare the inflammatory cell infiltration and epidermal degeneration [24, 25]. The rabbits were randomly divided into two groups; each was given either 1 ml physiologic saline or 0.5 mg/ml PEG-PCCL via auricular vein. Rabbits were killed by an overdose of chloral hydrate (4%, Sigma, USA) at 24 h after nanoparticles infusion. Two ear vein samples, including the region 10–15 mm from the catheter tip both proximally and distally, were obtained. These veins were fixed in 4% paraformaldehyde solution. Then paraffin cross-sections were prepared and stained with hematoxylin and eosin (HE). Histopathological examination was performed blindly. The findings were graded according to the criteria shown in table which were based on those of Kuwahara [17] with the addition of epidermal degeneration.

### Hepatorenal Function Tests

After 7 days of PEG-PCCL (0.5 mL, 20 mg/kg) administration on mice, the blood samples were extracted from the orbital venous plexus (2 mL) and immediately centrifuged at 1300 g, 4 °C. The supernatant was withdrawn. Then, the serum biochemistry parameters [26], including aspartate aminotransferase (AST), alanine aminotransferase (ALT), alkaline phosphatase (ALP), bilirubi, creatinine, uric acid, and albumin were evaluated using animal biochemical automatic analyzer (Dri-Chem 3000, Fuji Photo, Tokyo, Japan), which are indirect indicators of liver and renal function.

### Histopathological Examination

The histopathological changes of the lung, liver, and kidney were examined with H&E staining at 24 h, 48 h, and 7 days after injection of PEG-PCCL, PEI solution, or normal saline (0.5 mL, 20 mg/kg) via mice tail vein. These organs were obtained after animal sacrifice. Histopathology section and H&E staining were performed as described elsewhere [26]. The histopathological changes were observed under light microscope and were recorded with assorted camera (Leica, Co. Ltd., Germany).

### Blood Concentration

Blood concentration of PTX was calculated using spectrophotometer (LAMBDA 950, PerkinElmer, China). Blood samples were drawn from mice orbit at 0.08, 0.25, 0.5, 0.75, 1, 2, 6, 12, 24 h after treatment. Supernatant (100 μL) was collected after centrifuged at 1300 g for 10 min. The PTX concentration in each blood sample was determined with spectrophotometer at 760 nm.

### Mouse Tumor Models and Treatment

H22 cell suspension (0.25 mL, 4 × 10^6^ cells/mouse) was intraperitoneally injected into Balb/C mice at day 0. When the ascites formed (at day 3~5), tumor-bearing mice were divided randomly into three groups (*n* = 5) and treatment was initiated. The mice were injected with normal saline, PEG-PCCL/PTX (20 mg/kg), or PTX (10 mg/kg) intraperitoneally at a volume of 0.5 mL at days 3 and 10 to evaluate the anti-tumor efficacy. The abdominal perimeter (AP) was measured every day so as to calculate the increased percentage of AP (IPAP) as following formulation: IPAP = (*P*_n_ − *P*_0_)/*P*_n_. At day 10, the surviving mice were sacrificed by cervical dislocation, and the ascites were collected and weighed. Mice survival time was observed until day 20. Tumor-bearing mice were executed at the endpoint with distress signs, including high respiratory rate, fur ruffling, hunched posture, reduced activity, and progressive ascites formation [27]. The anti-tumor activity was evaluated comprehensively by survival days, abdominal perimeter, and the volume of ascites. The mice were fed under standard laboratory conditions.

### Statistical Analysis

Statistical analysis was performed with SPSS 19.0 (IBM, NY, USA). Every experimental treatment was repeated independently for at least three times. Data were presented as mean ± standard deviation (SD). Analysis of variance (ANOVA) was employed for statistical analysis. The grade of each phlebitis findings was analyzed by the Wilcoxon rank sum test. Dunnett’s test was used to compare the individual interventions. Statistical significance was indicated at *P* < 0.05.

## Results

### Morphology, Diameter, and Zeta Potential of PEG-PCCL

**Fig. 2:**
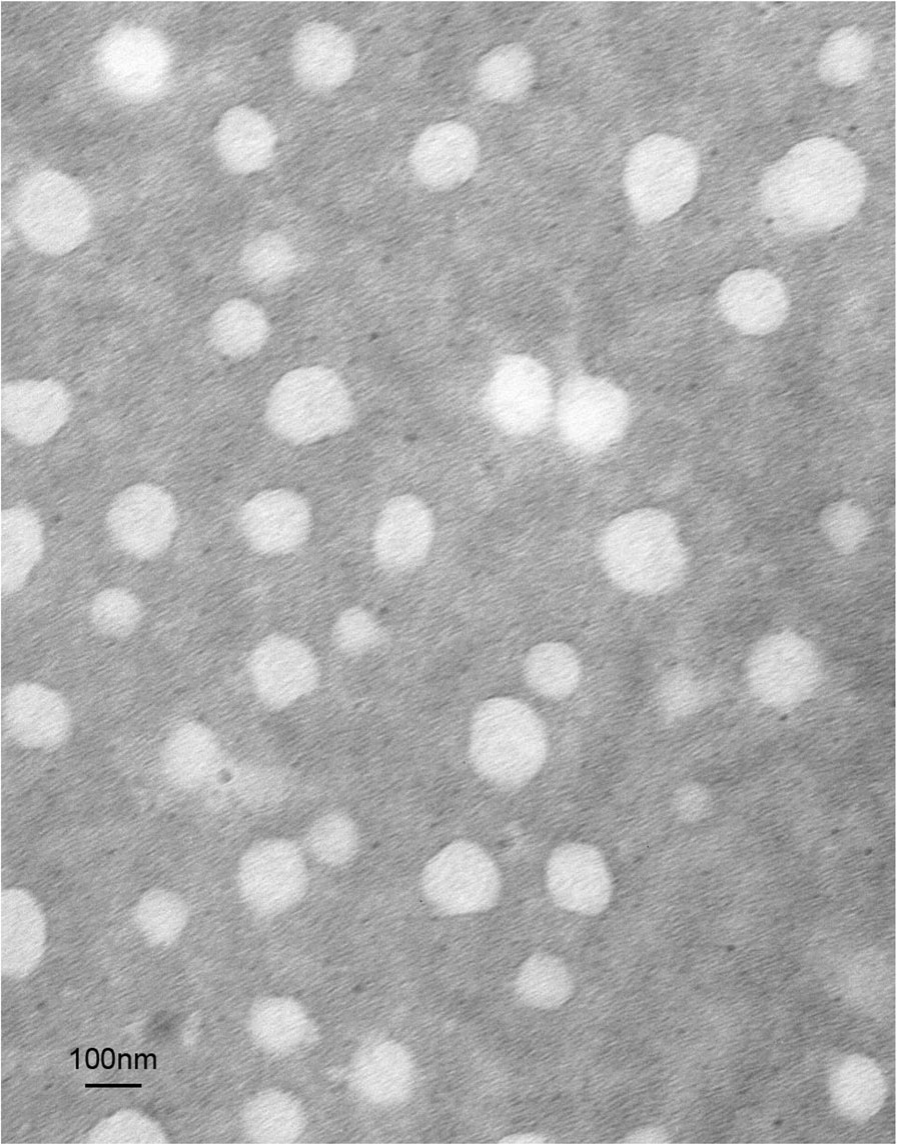
The characteristic of PEG-PCCL: TEM image. Scale bars were 100 nm

The results from the laser particle size analyzer showed that the average diameter of PEG-PCCL was 97 ± 2.6 nm. The TEM results of PEG-PCCL (Fig. 2) revealed that PEG-PCCL was spherical shapes and uniform in the solution. The average zeta potential of PEG-PCCL was − 18.4 mV. Minicolumn centrifugation method showed the EE% was 55.98%.

### Cytotoxicity

**Fig. 3:**
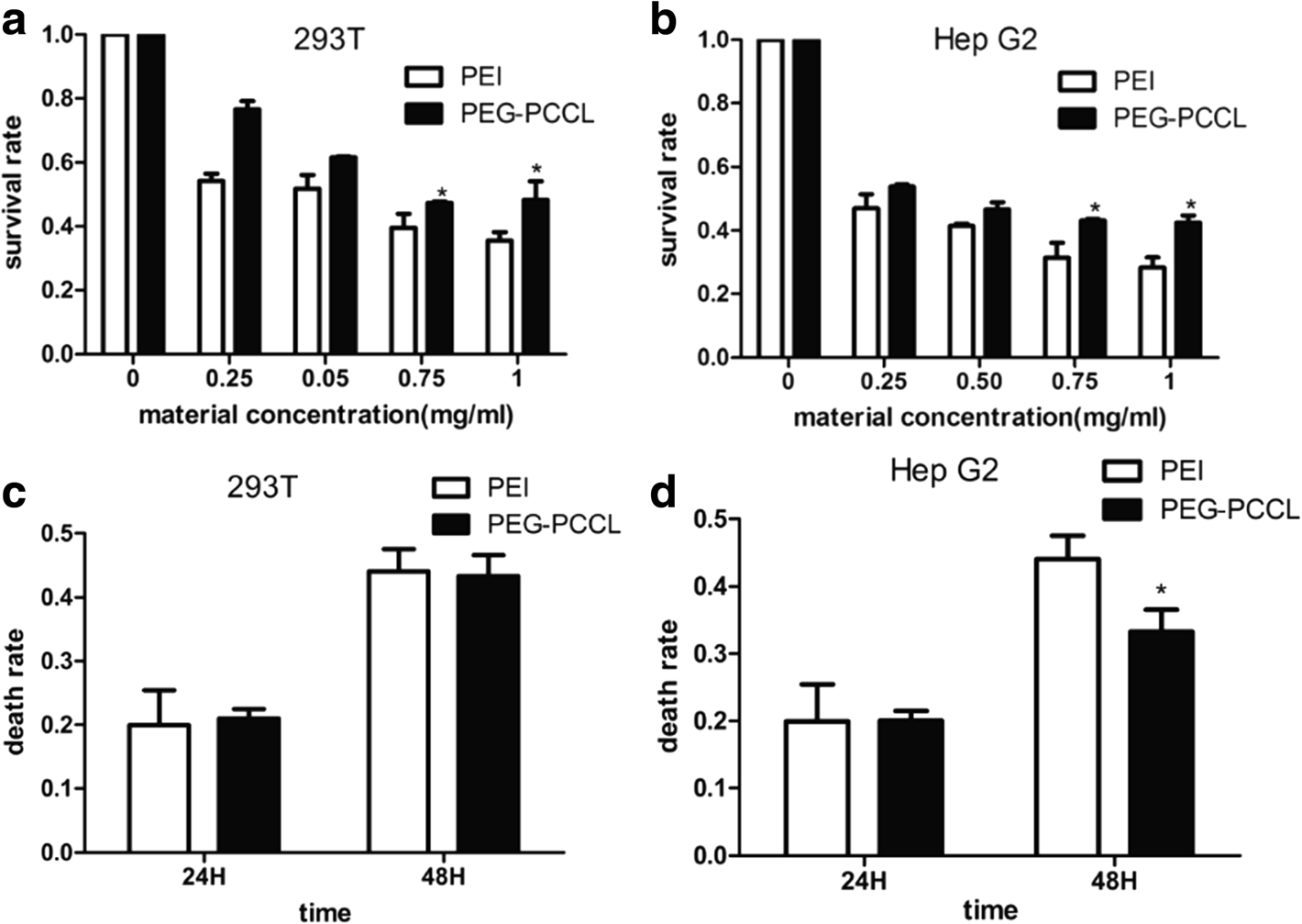
The cytotoxicity of PEG-PCCL compared with PEI. **a**, **b** MTT assay showed the survival rate of 293 T and HepG2 concentration dependently compared with negative controlled group(PEI). **c**, **d** LDH leakage assay of 293 T and HepG2 exposing to PEG-PCCL and PEI after 48 h. *P < 0.05 versus PEI group

The cytotoxicity of PEG-PCCL was evaluated by comparing to PEI, a typical nanoscale vehicle, in HEK293T and Hep-G2 cell lines. Within the concentration range (from 0 to 1 mg/mL) of PEG-PCCL or PEI, the viability of both HEK293T cells (Fig. 3a) and tumor cells (Hep-G2) (Fig. 3b) was descended in a concentration dependent manner. The embryonic cells appeared more sensitive at the concentration of 0.25 mg/mL, while tumor cells over 1 mg/mL. Compared to PEI, PEG-PCCL showed less cytotoxicity, especially at higher concentrations, i.e., 0.75 and 1 mg/mL (*P* = 0.023). LDH assay showed that the death rate in both embryonic cells and tumor cells was increased with exposing time; (i) it was 19 and 42% at 24 and 48 h respectively at 0.5 mg/mL PEG-PCCL (Fig. 3c) less than that of PEI. (ii) Tumor cells showed a slightly lower death rate (32%) at the time of 48 h when cells were exposed to PEG-PCCL in comparison to PEI (*P* = 0.037) (Fig. 3d).

#### SEM

**Fig. 4:**
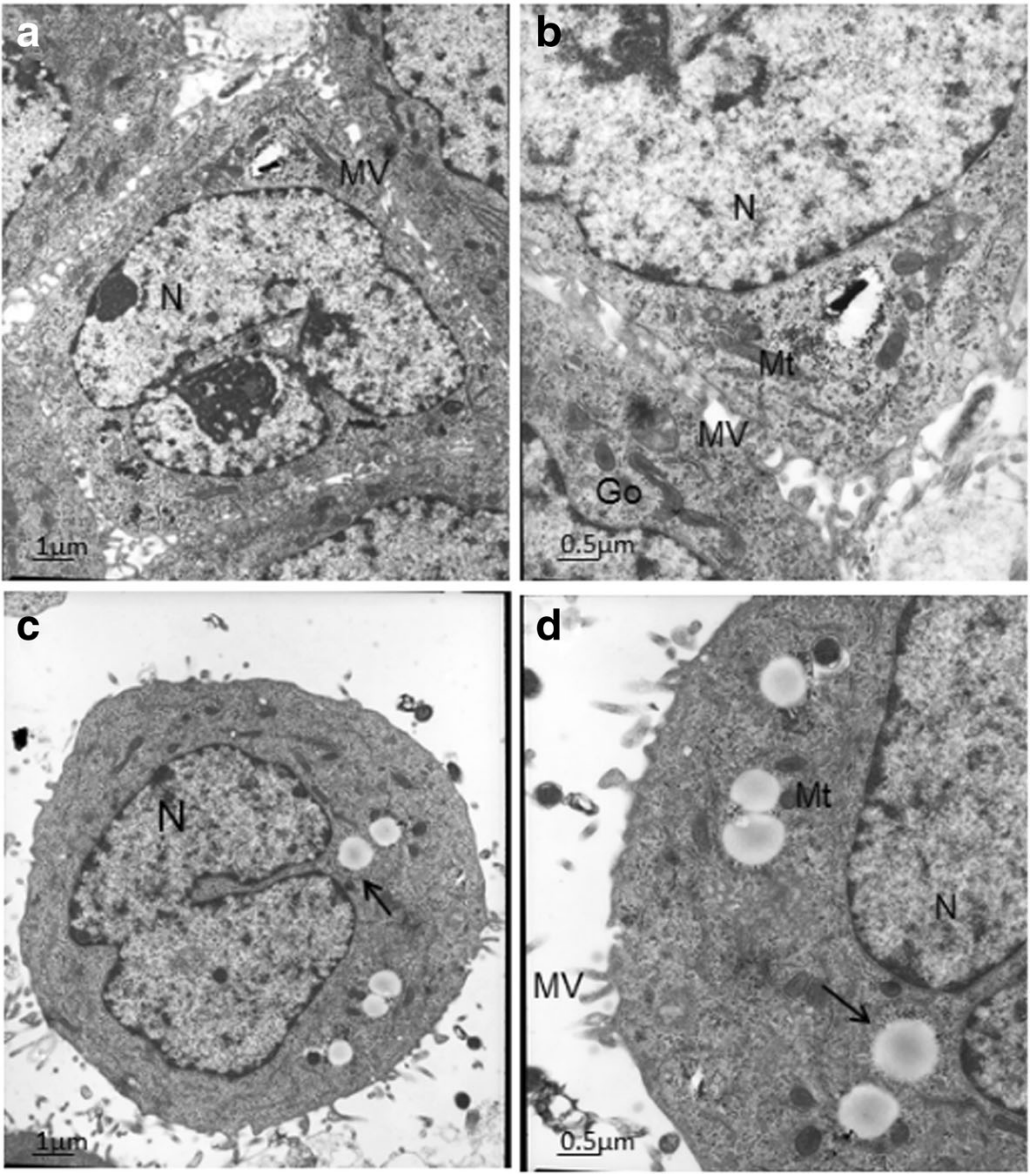
Electron micrographs. **a**, **b** Normal HepG2 cells. **c**, **d** Cells treated with PEG-PCCL nanoparticles through SEM (scanning electron microscope). The dark arrow points at the pinocytotic vesicles

The electron microscopic images of intact Hep-G2 cells and cells treated with PEG-PCCL nanoparticles were shown in Fig. 4. In intact Hep-G2 cells, there were abundant microvilli (Mv) at each surface, and the nucleus (N) was located more toward bases than apical portion. Numerous mitochondria (Mt), Golgi complex (Go), and rough endoplasmic reticulum (RER) were distributed in the cytoplasm. In PEG-PCCL-treated cells, the pinocytotic vesicles were definitely increased. No significant histopathological changes were observed, and round N and cytoplasmic organelles including Mt, RER, and Go were intact.

#### Apoptosis

**Fig. 5:**
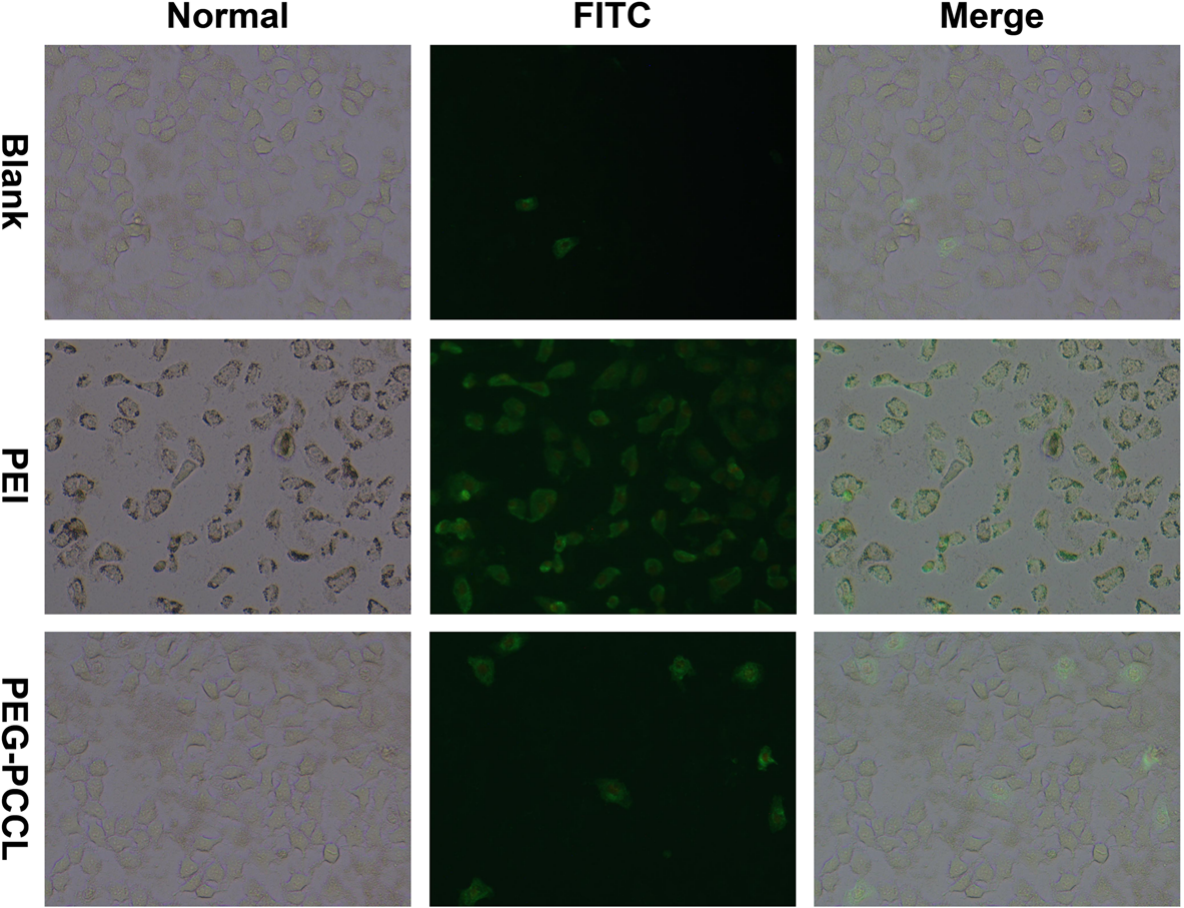
FITC-Annexin V stain represented cell apoptosis. HepG2 cells incubated with nanoparticles at a concentration of 0.5 mg/mL for 48 h and then co-stained with propidium iodide (red) and annexin V (green) were imaged at × 40

Significant decrease of cell viability may be correlated to the characteristics of particle size, surface chemistry, and concentration [28], which prompted our interest in the mechanisms underlying the observed cell death in HepG2 cells. To determine if the cell death was attributed to apoptosis or necrosis, HepG2 cells were treated with PEI or PEG-PCCL for Annexin V and PI co-staining. As observed under fluorescence microscope (Fig. 5), the cells treated with either PEI or PEG-PCCL showed earlier apoptosis compared to blank control group, as indicated by green fluorescence stained by annexin V. PEI-treated cells exhibited more potent apoptosis than those of PEG-PCCL, which was in line with previous result of MTT assay.

### Biocompatibility

#### Hemolysis

**Fig. 6:**
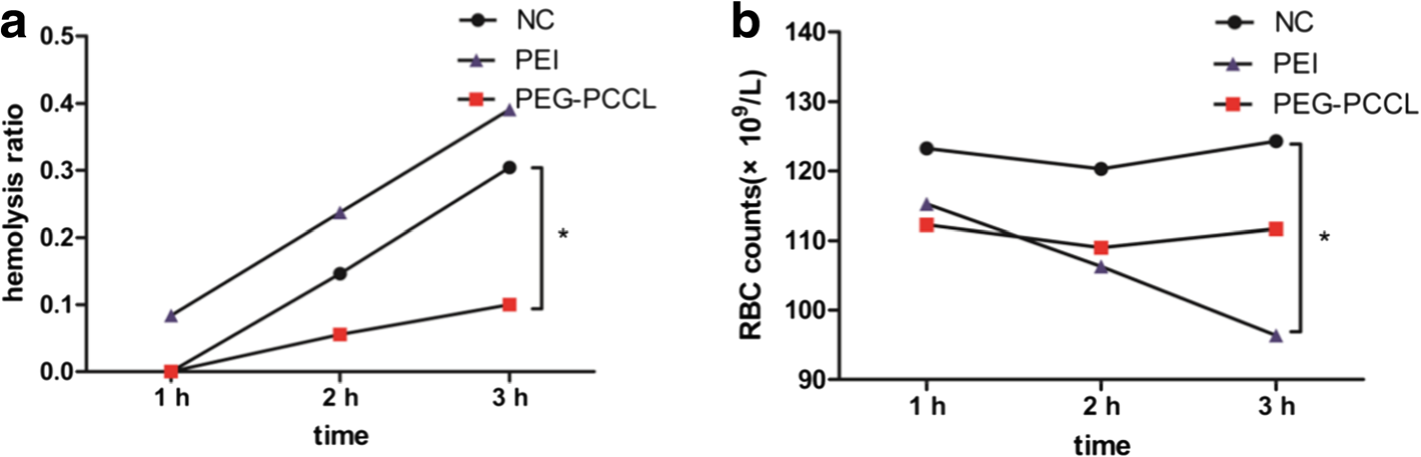
Hemolysis ratio and cell count (× 10^9^/L) of blood sample in 3 h. In vitro (**a**) in vivo (**b**) *P < 0.05 versus negative control group

As biocompatible nanoparticles are designed for purpose of endovascular applications, the investigations of hemocompatibility and endothelial cytotoxicity are required. In vitro test showed that, during the 3 h of observation, hemolysis increased with time, in which PEG-PCCL exhibited lower hemolysis ratio than normal saline (Fig. 6a). The in vivo test revealed the similar tendency. In contrast, PEI caused severe hemolysis both in vitro and in vivo (Fig. 6b).

#### Rabbit Phlebitis

**Fig. 7:**
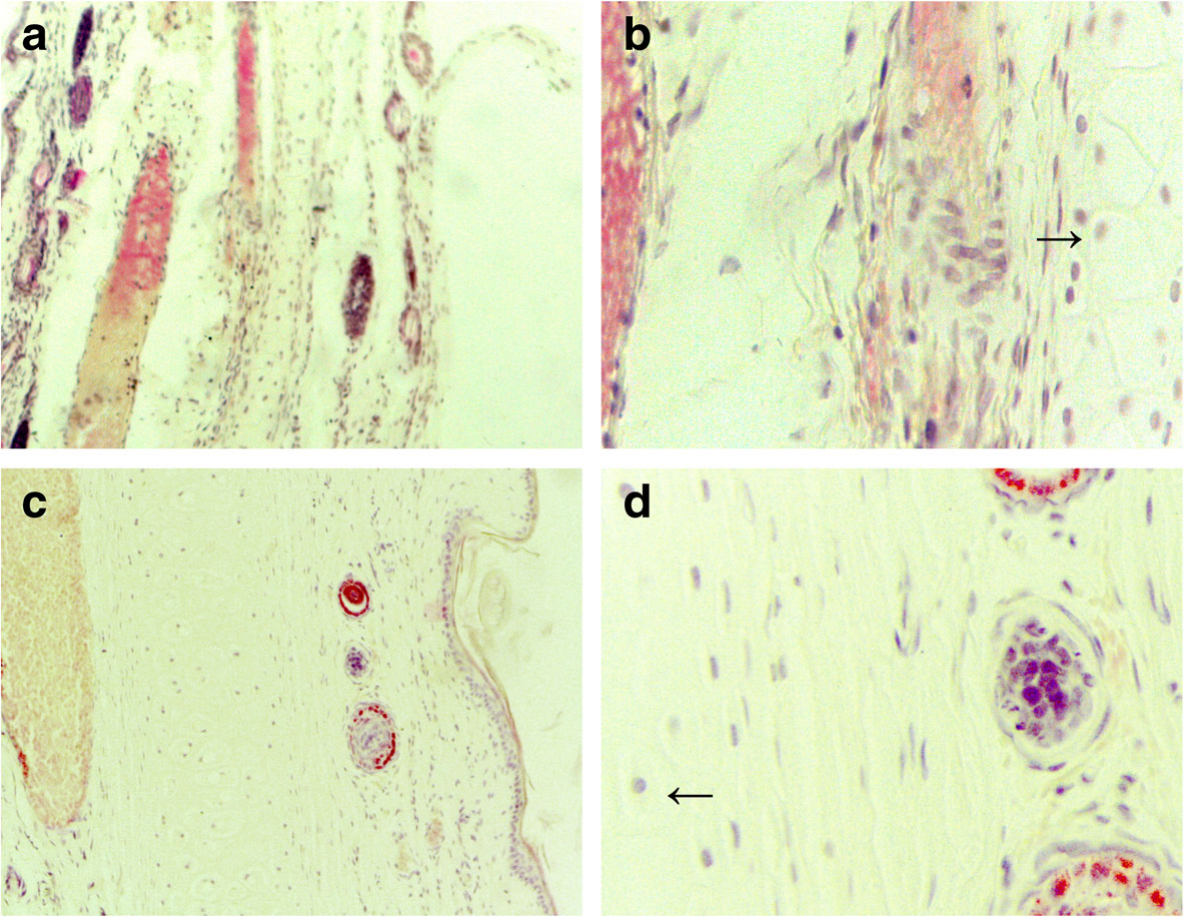
Photomicrographs of the ear vein after infusion of 24 h stained by H&E. **a**, **b** Group of infusion with normal saline. **c**, **d** Group of infusion with PEG-PCCL. The needles represent the auricular cartilage. (Left image × 10, right image × 40)

The histopathological examination (Fig. 7) illustrated intact vascular endothelium without chrondrocyte necrosis in the auricular cartilage. Little edema of the proximal part of the vein was observed. In the respect of the loss of venous endothelial cells and inflammatory cell infiltration, co-infusion (Table 1) of groups treated with PEG-PCCL after 12 and 24 h tended to be increased, but the improvement was not statistically significant (*P* > 0.05).

**Table 1:** Effect of post-infusion with PEG-PCCL on the histopathological grade in 6 rabbits at 12 and 24 h

		Inflammatory cell infiltration				*P* value	Edema				*P* value	Thrombus				*P* value	Epidermal degeneration				*P* value
	Grade	0	1	2	3		0	1	2	3		0	1	2	3		0	1	2	3	
Group	Control	6	0	0	0		4	2	0	0		6	0	0	0		5	1	0	0	
	Post-infusion with PEG-PCCL (12 h)	5	1	0	0	N.S.	4	1	1	0	N.S.	6	0	0	0	N.S.	4	2	0	0	N.S.
	Post-infusion with PEG-PCCL (24 h)	5	1	0	0	N.S.	4	2	0	0	N.S.	6	0	0	0	N.S.	4	1	1	0	N.S.

#### Organs Toxicity

**Fig. 8:**
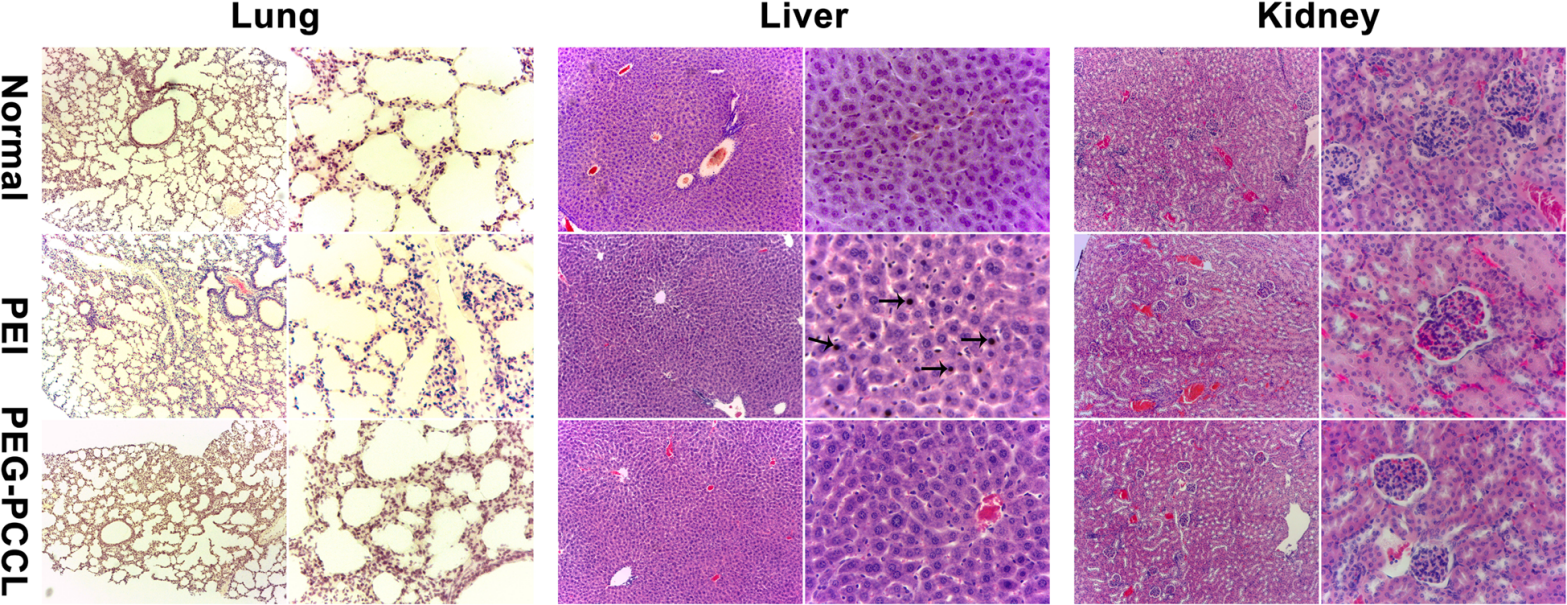
The H&E dyeing light microscopic images of the lung, liver, and kidney. The images are collected from mice intravenous administrated with NS, PEI, and PEG-PCCL. The needles represent the karyopyknosis of hepatocyte. (Images of left columns, × 10; images of right columns × 40)

To evaluate the acute toxicity of PEG-PCCL in major organs, histopathological examination in the lung, liver, and kidney was performed after intravenous administration of PEG-PCCL at 0.5 mg/mL for 3 days in mice (*n* = 5). Normal saline and PEI were used as controls. The results showed that PEI caused slight inflammation and lobular interstitium thickness and hepatocyte karyopyknosis (Fig. 8). Although, compared to normal saline group, no apparent histopathological changes were observed in all examined organs in PEG-PCCL-treated group (Fig. 7); hepatorenal function was examined for further confirmation of its nontoxicity (Table 2).

**Table 2:** The hepatorenal function of mice treated with PEG-PCCL (mean ± SD, *n* = 2)

Samples	AST (U/L)	ALT (U/L)	ALP (U/L)	Albumin (g/L)	Creatinine (μmol/L)	Uric acid (μmol/L)
NS	17.50 ± 3.54	4.00 ± 0.00	23.00 ± 9.90	6.35 ± 0.07	5.50 ± 0.71	15.50 ± 6.36
PEG-PCCL	13.50 ± 0.71	5.00 ± 0.00	20.50 ± 7.78	4.75 ± 0.35	2.00 ± 0.00	18.00 ± 7.07

*Note*: data were collected 7 days after the administration of PEG-PCCL

### Pharmacokinetic Study

**Fig. 9:**
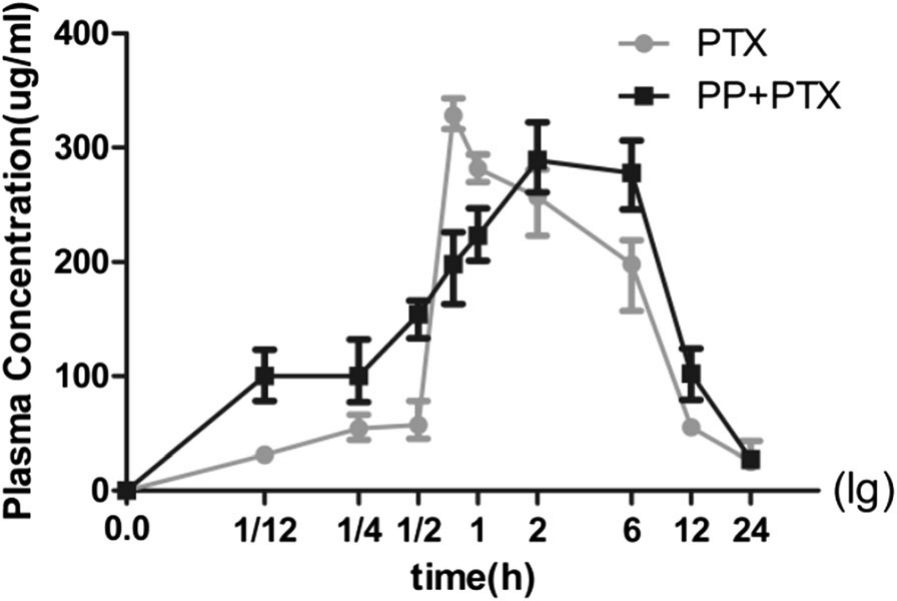
The blood concentration–time curve. In mice after intravenously administered with PTX or PP + PTX. (*n* = 6)

Pharmacokinetic study was performed after intravenously injection of 10 mg/kg PTX of Taxol® or 20 mg/kg PEG-PCCL/PTX (PP + PTX) (50% loading ratio). The peak of plasma concentration (Cmax) was 312 ± 2.59 μg/mL (PTX) and 283 ± 2.79 μg/Ml (PP + PTX). The time of maximum concentration (Tmax) and the area under the plasma concentration-time curve were 0.54 ± 0.20 h, 52.00 ± 4.30 μg h/mL and 4 ± 1.22 h, 282.21 ± 21.08 μg h/mL for PTX and PTX-NPs, respectively. The blood concentration-time curve was shown in Fig. 9.

### Effect of PEG-PCCL/PTX on Tumor-Bearing Mice

**Fig. 10:**
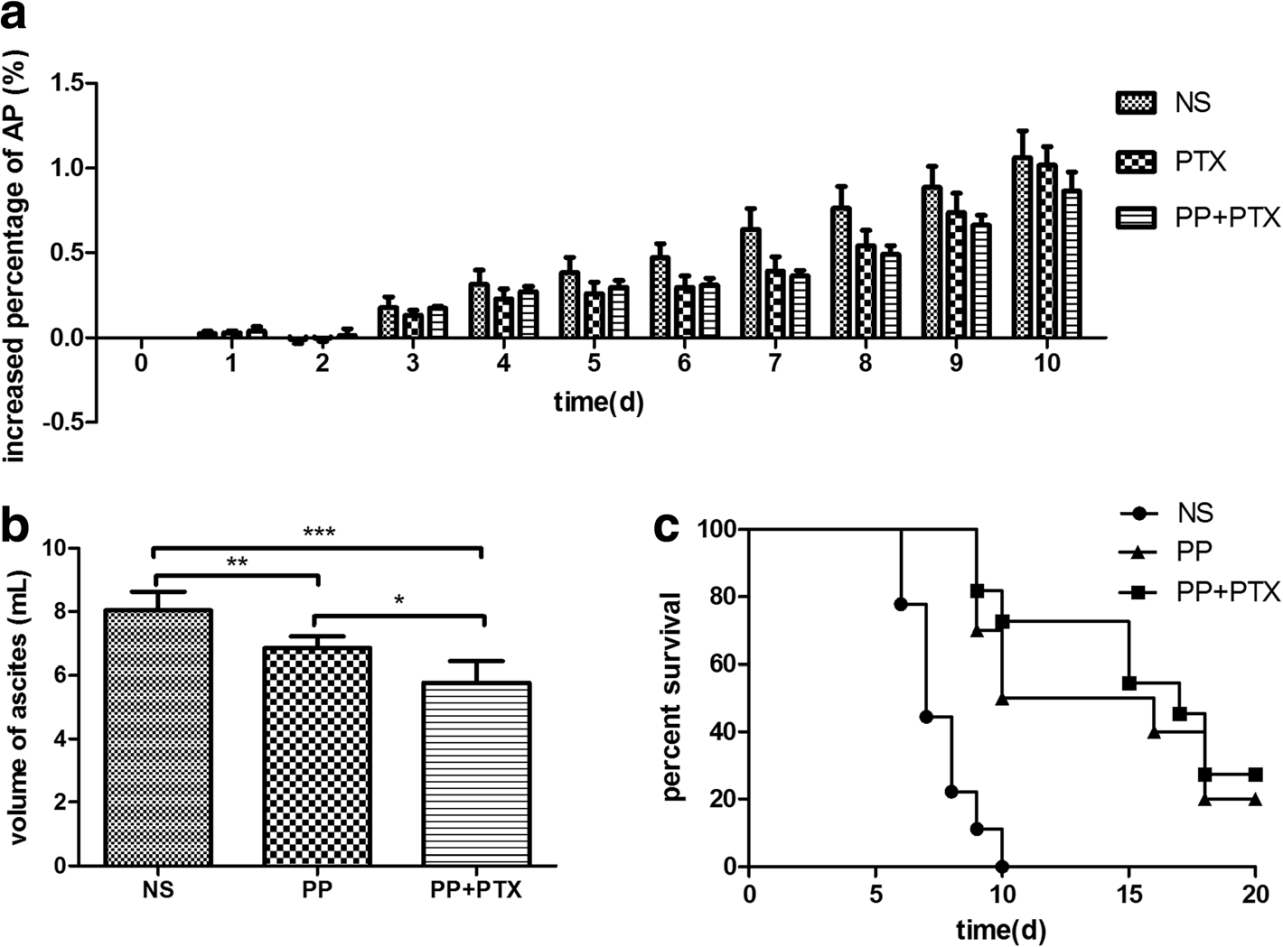
The anti-tumor effect of PP/PTX in H22 tumor-bearing mice. **a** Balb/C mice (*n* = 5) were intraperitoneally injected with PP/PTX or PTX at day 3. **b** Ascites of each group (*n* = 5) were collected at day 10. **c** The survival of each group (*n* = 10) was observed daily. *P < 0.05, **P < 0.01, ***P < 0.001

To investigate anti-tumor effect of PEG-PCCL/PTX in vivo, (i) the increased percentage of abdominal perimeter (IPAP) of H22 tumor-bearing mice was calculated daily (Fig. 10a). At day 3, the ascites started to form and the IPAP of each group was dramatically raised, in which PP/PTX group and PTX group, compared to NS group, showed slower increase with time. (ii) Ascites was collected at day 10, and the volume was measured (Fig. 10b). Compared with NS group, the volume of ascites of both PTX group and PP/PTX group was significantly reduced (*P* = 0.0005 and *P* = 0.0052), where PP/PTX group exhibited a lower volume than PTX group (*P* = 0.0138). (iii) the survival of each group was observed for 20 days from day 0. PP/PTX group and PTX group had a longer lifespan and higher surviving rate than NS group.

## Discussion

Many polymers, including PEI, have been suggested to be carriers of chemical and biopharmaceutical drugs for an appropriate conjugation. PEI is a widely investigated cationic polymer and has been considered the gold standard in assays regarding transfection efficiency [29]. However, the side effects (e.g., cytotoxicity) impede PEI to be applied in medical use. To search for a safer and effective nano-based material, we have successfully prepared a novel candidate of drug delivery; PEG-PCCL is characterized by high hydrophilicity and favorable stability due to the effect of the hydrogen bond. The main purpose of the study was to assess the in vivo and in vitro toxicity of PEG-PCCL, which opens potentially therapeutic window for treating cancer.

Since enhanced permeability and retention effect is considered as tumor-selective delivery mechanisms, the nano-sized drugs have been paid considerable attentions [30, 31]. The nanoparticles with smaller diameter have better immunocompatibility [32] and less uptake by the liver, longer blood circulation time, and higher bioavailability [33].Our dipolymers are of appropriate sizes (97 ± 2.6 nm) (Fig. 2) to permeate into and out of the tumor blood vessel as well as evade the host immune system, while the cationic polymers with the positive zeta potential were reported to be toxic [34]. This toxicity of PEG-PCCL with negative zeta potential was supposed to be avoided ideally. Indeed, in the toxicity assessments in the present study, there was little toxicity concerns with PEG-PCCL. Similar results were also reported by other lab [35–37].

The in vitro experiments revealed a toxicity-free property for PEG-PCCL. As observed under SEM (Fig. 4), (i) pinocytotic vesicles recognized by dark arrow indicated a liable endocytosis [38]. (ii) Complete nucleus and intact organelles (Mt, RER, and Go) showed little cytotoxicity and good biocompatibility of PEG-PCCL at the cellular level. In the MTT assay and LDH leakage assay (Fig. 3), PEI and PEG-PCCL led to similar trend of cell viability in a dose- and time-dependent manner in both 293 T cells and HepG2 cells though, PEG-PCCL exhibited lower cytotoxicity especially at a higher concentration (*P* < 0.05). These results suggested that the covalent binding of carboxyl does not increase extra toxicity. In vitro hemolysis is widely accepted as a useful and reliable method for evaluating NP blood compatibility. In in vitro hemolysis test, PEG-PCCL showed lower hemolysis ratio than normal saline (Fig. 6a). This may be due to the negative potential of PEG-PCCL that protects the blood cells. The in vivo hemolysis test revealed a similar tendency. In contrast, PEI exhibited severe hemolysis. Collectively, PEG-PCCL nanoparticles are hemocompatible as they did not exhibit any hemolytic effect neither in vitro nor in vivo model (Fig. 6b).

The innoxious nature of PEG-PCCL was further proved by in vivo experiments. Since phlebitis induced by intravenously administered antineoplastic agents [24] is frequently seen in clinical practice, we tested the inflammatory reaction after PEG-PCCL injection. In the rabbit phlebitis study (Fig. 7), little inflammatory infiltration or tissue edema was observed, indicating that the new formulation of chemotherapeutic drugs captured by PEG-PCCL is biocompatible, and that co-infusion of PEG-PCCL could be a favorable candidate as intravenous drug deliverer. Meanwhile, in the H&E staining assay (Fig. 8), no obvious histopathological changes of organs were observed in PEG-PCCL nanoparticles treatment group compared to normal group. Further hepatorenal function was investigated for safety evaluation (Table 2), in which no significant differences were shown between normal saline group and PEG-PCCL group. Consequently, it is evident that PEG-PCCL is a kind of safe and non-toxic nanoparticles with potentiality to be applied in targeting intervention.

Furthermore, preliminary evaluation of drug-loaded effect in regard of pharmacokinetics and anti-tumor was carried out in H22 tumor-bearing mice. In the pharmacokinetic study (Fig. 9), Tmax of PP + PTX was 4 ± 1.22 h, which exhibited better persistence than that of PTX (0.54 ± 0.20 h). PP + PTX also performed larger area under the plasma concentration-time curve than PTX. These results showed that PEG-PCCL/PTX lasted longer than PTX alone in blood, which indicated higher stability and more delayed release of PEG-PCCL/PTX. However, the EE% of PEG-PCCL was dissatisfactory (55.98%) and sustained drug release was less than ideal (4 ± 1.22 h). Therefore, more research is needed to modify the nanoparticle to achieve a higher EE and longer drug release. For example, adjusting the proportion of PEG and PCCL can be considered [39]. Although no metastasis was seen in abdominal organs in our model, tissue distribution and concentration of PTX-NPs should be investigated in further study to find out the possible side effects. Moreover, the combination of PEG-PCCL and PTX made an improvement in life expectancy and a reduction in tumor ascites formation (Fig. 10). Notably, the anti-tumor effect was enhanced when PTX was loaded with PEG-PCCL, suggesting a possibility of a promising drug carrier. Besides, nanoparticles may be beneficial to confront anti-tumor drug resistance. Modification with folate [40] has been reported to overcome TLR4 driven chemotherapy resistance, and its co-encapsulation of anti-tumor agents [41] could be a promising option. Nanoparticle modified by additional Fe_3_O_4_ [42] may be more easily guided to its targets under the applied magnetic field, which would lower chemotherapeutic drugs-induced systemic toxicity [43].

## Conclusions

PEG-PCCL nanospheres showed less cytotoxicity and better biocompatibility than mature medical nanoparticles (PEI) at the therapeutical concentration. PEG-PCCL-loaded PTX revealed higher stability and slower release in tumor mice. These results suggest that PEG-PCCL is a potential candidate of biocompatible drug vehicle for hydrophobic drugs.
